# Biocomposite Material Based on *Lactococcus lactis* sp. Immobilized in Natural Polymer Matrix for Pharmaceutical Removal from Aqueous Media

**DOI:** 10.3390/polym16131804

**Published:** 2024-06-26

**Authors:** Narcis-Teodor Niță, Elena-Mirela Suceveanu, Florin Marian Nedeff, Ovidiu Tița, Lăcrămioara Rusu

**Affiliations:** 1 Doctoral Studies School, “Vasile Alecsandri” University of Bacau, 157 Calea Mărăşeşti, 600115 Bacău, Romania; narcisteodor170@gmail.com; 2 Faculty of Engineering, “Vasile Alecsandri” University of Bacau, 157 Calea Mărăşeşti, 600115 Bacău, Romania; mirela.suceveanu@ub.ro (E.-M.S.); florin_nedeff@ub.ro (F.M.N.); 3 Faculty of Agricultural Sciences, Food Industry and Environmental Protection, “Lucian Blaga” University of Sibiu, Doctor Ion Rațiu, No.7, 550012 Sibiu, Romania; ovidiu.tita@ulbsibiu.ro

**Keywords:** biosorption, calcium alginate, equilibrium isotherms, ethacridine lactate, immobilization, kinetic models, *Lactococcus lactis*

## Abstract

Ecosystems are negatively impacted by pharmaceutical-contaminated water in different ways. In this work, a new biosorbent obtained by immobilizing *Lactococcus lactis* in a calcium alginate matrix was developed for the removal of pharmaceuticals from aqueous solutions. Ethacridine lactate (EL) was selected as the target drug. Lactococcus Lactis biomass was chosen for the biosorbent synthesis for two reasons: (i) the microbial biomass used in the food industry allows the development of a low-cost biosorbent from available and renewable materials, and (ii) there is no literature mentioning the use of *Lactococcus Lactis* biomass immobilized in natural polymers as a biosorbent for the removal of pharmaceuticals. The characterization of the synthesized biosorbent named 5% LLA was performed by scanning electron microscopy (SEM) and Fourier transform infrared spectroscopy (FTIR) analysis. Additionally, particle size and the point of zero charge were established. Batch biosorption investigations showed that using 5% LLA at an initial pH of 3.0 and a biosorbent dose of 2 g/L resulted in up to 80% EL removal efficiency for all EL initial concentrations (20–60 mg/L). Four equilibrium isotherms, given in the order of Redlich–Peterson > Freundlich > Hill > Temkin, are particularly relevant for describing the experimental data for EL biosorption on the 5% LLA biosorbent using correlation coefficient values. Kinetic parameters were determined using kinetic models such as pseudo-first-order, pseudo-second-order, Elovich, Avrami and Weber–Morris. The pseudo-second-order kinetics model provides the greatest fit among the evaluated equations, with correlation coefficients greater than 0.99. According to the study’s findings, the developed biocomposite is a potentially useful material for the removal of pharmaceuticals from aqueous matrices.

## 1. Introduction

Recent years have seen an increase in reports of emerging contaminants (ECs) in the environment from all over the world, mostly as a result of the growth of the industrial and medical sectors [1,2]. A well-known class of ECs is represented by pharmaceuticals whose presence in the environment is of significant concern to the health of humans and members of terrestrial and aquatic ecosystems [2,3,4,5].

Pharmaceutical products, which are critical to the sustainability and maintenance of human health, are produced and used in ever-increasing quantities, resulting in their emergence as fast-growing pollutants, as evidenced by the presence of their residues in all environmental matrices from every continent over the last 30 years [5,6,7,8,9,10,11]. These include surface water (lakes, rivers, streams and seawater), groundwater, effluents and influents from wastewater treatment plants (WWTPs), sediments and sludge [5,12,13,14]. They now occur widely in the geosphere and biosphere and even in the polar regions, considered as the cleanest environment on Earth [15,16,17,18,19]. These are natural or synthetic substances that contain polar molecules with numerous ionizable groups, each with a distinct structure and function, and are either lipophilic or moderately soluble in water [20,21,22]. They are also divided into 24 therapeutic classes, with about 10,000 different pharmaceutical products containing 3000 to 4000 different active substances [23]. Among the products on the market, the most consumed are antibiotics, anti-inflammatories, analgesics, antidepressants, anti-epileptics, hypolipidemics, *β*-blockers, anti-ulcer drugs, antimicrobials, antiseptics and antihistamines [9].

The persistence of pharmaceuticals in the environment, combined with their widespread and increasing use and production, resulting in continuous environmental input, even at low concentrations (from µg/L to ng/L), and potential toxicological effects on non-target organisms, has become a concern for the scientific community [2,3,24,25]. These contaminants can cause endocrine disruption, change the composition and essential functions of natural microbial communities, negatively impact fish and invertebrates and, in the case of antibiotics, contribute to the emergence of antibiotic-resistant genes and bacteria, according to experimental studies [20,26,27].

The main sources of pharmaceutical contamination of surface water, groundwater and drinking water include effluents from the pharmaceutical industry, hospitals and wastewater treatment plants, uncontrolled leachate from landfills and incorrect disposal of drugs [4,20,21]. The key problem is that wastewater treatment plants (WWTPs) are unable to entirely degrade drugs since they are usually designed to handle easily and moderately degradable organics in mg/L levels. This can be explained by two factors: firstly, pharmaceutical products have a wide range of solubility, absorbability, volatility, biodegradability, polarity and stability values; secondly, they can be present and active at very low concentrations (ng/L–μg/L) [5,8,28]. As primary and secondary WWTP treatments are generally not able to remove these pollutants, leading to their migration into drinking water supplies, there is a need to create advanced and efficient tertiary treatment processes that allow the total removal of pharmaceutical residues from wastewater before its release into the environment, an aspect highlighted in numerous scientific works [2,4,5,21,29]. 

A broad spectrum of methods has been used to remove pharmaceuticals from aqueous media, including membrane separation, ozonation, flocculation, advanced oxidation processes, photocatalysis, microbial degradation, electrochemical processes and adsorption [5,21,30,31]. These techniques differ in terms of effectiveness, durability, cost and other factors, and they are based on physical, chemical or biological processes, with a variety of advantages and disadvantages [2,5,21,31]. Among these, adsorption and advanced oxidation processes (AOPs) are two methods for successfully removing pharmaceutical compounds from water and wastewater. AOPs generate a large number of oxidation and transformation byproducts, some of which are more hazardous than the original chemical. In contrast, adsorption does not produce new products, giving it a substantial advantage over other processes [5,32]. Although the most promising method for drug removal from aqueous solutions is adsorption, it is largely applied at the laboratory level or implemented on a small scale [5,21]. Activated carbon is the most widely used adsorbent due to its versatility and efficiency. However, its high cost makes it unsuitable for sorption [33,34]. Research is therefore required to find more efficient and affordable pharmaceutical removal technologies that should be developed for rapid, large-scale implementation at low cost.

Biological materials, often known as biomass, are becoming increasingly relevant absorbent alternatives in this setting. According to the existing information and our previous studies, biomass can be either living or dead, and employing it as a biosorbent would enhance the quantity of contaminants that could be eliminated [30,35,36,37]. As a result, different fungal, bacterial, yeast and microalgal species have been investigated for their potential to remove various types of pollutants, with promising results [21,35,37,38]. The microbial biomass can be added directly or rendered immobilized or encapsulated in several matrices, including natural polymers (i.e., alginate, chitosan) among others [21,30,37]. 

Entrapment techniques (immobilization, encapsulation) allow for the cost-effective and easy removal of microbial biomass from wastewaters while also increasing mechanical resilience. In this regard, research on biosorption techniques is now focusing on the development of increasingly complex systems using innovative biocomposite materials [21]. 

*Lactococcus lactis* is a mesophilic lactic acid bacterium (LAB) and one of the most common starting cultures that plays a major role in dairy fermentations both commercial and artisanal. These are Gram-positive, catalase-negative, facultatively anaerobic, non-motile and non-spore-forming bacteria. The species currently comprises four subspecies, *lactis, cremoris, hordniae* and *tructae*, as well as one biovariant, *L. lactis* subsp. *lactis* biovar. *diacetylactis* [39]. 

Even though there are few studies, lactic acid bacteria have been recognized in the field of bioremediation, especially when the bacterial strains act as adsorbents [40,41,42]. The literature presents some studies on the removal of metals by biosorption using these bacteria. For example, *Lactobacillus acidophilus* is able to remove arsenic (III) from wastewater [43]. Halttunen et al. [44] showed that cadmium and lead ions can be bound to certain species of *Lactobacillus* and *Bifidobacterium*. Mishra et al. [45] investigated *Lactobacillus* as potential bacteria for bioremediation and found that chromium-resistant *Lactobacillus* strains can reduce chromium (VI) to chromium (III). Furthermore, *Lactobacillus casei* was discovered to be the most effective Cu^2+^ binding agent among the microorganisms studied [46]. In addition, Lin et al. [47] studied the mechanism of silver cation biosorption by *Lactobacillus* sp. strain A09. Also, Milanowski et al. [48] investigated the biosorption of silver cations by *Lactococcus lactis* and *Lactobacillus casei* bacteria isolated from dairy products. When the above factors are considered, it becomes clear that this biomass appears to meet all of the requirements for use in the production of a viable biosorbent: it is safe, low-cost and available in large quantities throughout the year, given that it can be obtained through biotechnological processes from microorganisms.

Ethacridine lactate (EL) (2-ethoxy-6,9-diaminoacridine monolactate monohydrate) is an acridine compound with antibacterial and antiseptic properties that is used to treat inflammatory or ulcerative dermatological disorders caused by Gram-positive bacteria and enteric disorders (i.e., diarrhea, shigellosis) [49,50]. According to the Occupational Safety and Health Administration Organization in the United States, EL is a hazardous substance having acute and chronic health impacts, as well as high toxicity for aquatic life [51].

Given that our previous study [52] demonstrated the ability of *Saccharomyces pastorianus* biomass immobilized in calcium alginate to retain pharmaceutical pollutants from aqueous solutions, which belongs to the fungi category, the question was raised whether other species of microorganisms currently used in the food industry, for example, lactic acid bacteria, could achieve promising results for the aforementioned purpose. The selection of a species of microorganisms that are used in the food industry allows for the development of a low-cost biosorbent from available and renewable materials, as these types of microbial biomass are abundant and inexpensive.

To the best of our knowledge, there is no literature mentioning the use of immobilized or encapsulated *Lactococcus lactis* biomass in natural polymers as a biosorbent for the removal of pharmaceuticals from aqueous solutions.

Therefore, the current study proposes the synthesis of a biocomposite material by immobilizing *Laccococcus lactis* (lactic bacteria) in a calcium alginate matrix and testing it for the removal of pharmaceuticals from aqueous media by biosorption. Ethacridine lactate was chosen as the target pharmaceutical compound. In order to remove EL from aqueous solutions in a batch system, this study set out to assess the biosorption capabilities of the developed biosorbent. Additionally, it used a mathematical approach to validate the experimental results. To describe the experimental data, several types of adsorption isotherms and kinetic models were studied. In addition, the equilibrium and kinetic parameters of the EL biosorption process were determined and discussed. 

This information will help to extend the database of biocomposite materials derived from renewable resources that can be utilized in biosorption processes to remove persistent organic pollutants such as pharmaceuticals, and it will also serve as the basis for progressing to the next technological level of testing (dynamic system).

## 2. Materials and Methods

### 2.1. Reagents and Analytical Procedure

The reagents required for the investigations were of analytical purity and had not been treated or purified.

**Figure 1:**
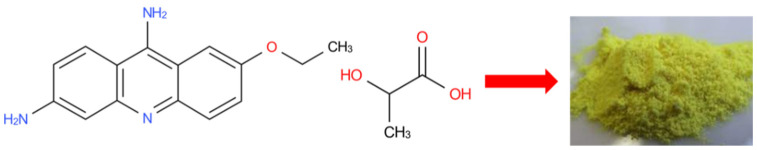
Ethacridine lactate (EL) structure (CAS 1837-57-6; molecular formula: C_18_H_21_N_3_O_4_; MW = 343.4 g/mol).

Ethacridine lactate (Figure 1) was supplied by Merck (Darmstadt, Germany). Chemical Company (Iași, Romania) provided hydrochloride acid, sodium chloride and ethanol. Chempur at Piekary Ślaskie, Poland, provided sodium hydroxide and calcium chloride. BUCHI Laboratortechnik AG (Flawil, Switzerland) supplied low-viscosity-grade sodium alginate.

The *Lactoccocus lactis* biomass used in the experiments was a commercial lactic culture (Danisco Choozit MA016), containing freeze-dried concentrated lactic ferments, for the direct inoculation of milk, provided by Danisco Company (Copenhagen, Denmark). The commercial product contains two subspecies of *Lactococcus lactis*: *Lactococcus lactis* ssp. *lactis* and *Lactococcus lactis* ssp. *cremoris.*

All the solutions that were used in the experiments were prepared with distilled water. pH adjustments were carried out using NaOH (0.1 M) or HCl (0.1 M).

A stock solution of EL (500 mg/L) was prepared and stored in a closed vessel at 4 °C. Subsequent dilutions (1 mg/L to 70 mg/L) were made, and their absorbance was measured at 431 nm using a UV1280 spectrophotometer (Shimadzu, Tokyo, Japan) to plot the calibration curve. All experiments were performed in triplicate.

### 2.2. Biosorbent Synthesis and Characterization

#### 2.2.1. Synthesis of Biosorbent Using Biomass of *Lactococcus lactis*

Before the synthesis of the biosorbent, the *Lactococcus lactis* biomass was inactivated by drying the commercial culture at 90 °C for 2 h. Inactivated *Lactococcus lactis* biomass was added to a sodium alginate solution (1%) in order to form a suspension with a concentration of 5% (d/w). The mixture was thoroughly homogenized before it was dropped into a 2% calcium chloride solution. The resulting beads (called 5% LLA) were washed with 2% CaCl_2_ and stored in an identical fresh solution for 48 h at 4 °C before their first use.

Before starting each biosorption experiment, the storage solution was removed, and the beads were washed with distilled water.

#### 2.2.2. Biosorbent Characterization (SEM, FTIR, Point of Zero Charge)

*SEM analysis.* The scanning electron microscopy (SEM) investigation was carried out using a SEM Quanta 200 3D (FEI Europe B.V., Eindhoven, The Netherlands) apparatus with an energy-dispersive X-ray system. Prior to this experiment, the biosorbent beads were dried at 50 °C for 2 h in an Air Performance AP60 hot air oven (Froilabo, Paris, France) before being placed in stubs with double adhesive carbon discs. The typical secondary electron mode (SE) in low vacuum was utilized. A large field detector (LFD) with a 20 kV accelerating voltage, a working distance of 14.6–15.5 mm, and a spot size of 5 was used to ensure detection. The magnification range was 1 mm to 10 μm.

*FTIR analysis.* FTIR spectra were recorded between 4000 cm^−1^ and 400 cm^−1^ (32 sample/background scans; 4 cm^−1^ resolution) using an IRSpirit-X Series FTIR spectrometer (Shimadzu, Tokyo, Japan) coupled with an ATR. Following each spectrum, the ATR was cleaned with ethanol. The reference background spectrum was obtained using air. 

*Point of zero charge determination*. In order to determine the point of zero charge (pH_PZC_) value, 0.4 g of biosorbent was mixed for 24 h on an orbital shaker using an SK-O180-S Digital Orbital Shaker device (DLAB SCIENTIFIC Company, LTD., Beijing, China) at room temperature with 20 mL of 0.1 M NaCl solutions with an initial pH of 2 to 12. A portable pH meter (Dostmann KLH9.1, 0–14 pH, Carl Roth, Germany) was utilized for initial (pH_i_) and final (pH_f_) pH measurements. In the following step, a plot was generated using the collected data.

### 2.3. Batch Biosorption Methodology

The effects of the key parameters (pH, biosorbent dosage and initial EL concentration) on the biosorption process were investigated. The experimental setting began by investigating the influence of the initial pH of EL solutions (40 mg/L). Its value varied from 2 to 12, and a biosorbent dose of 2 g/L was used. The evaluated biosorbent doses ranged from 1 to 5 g/L. The increase in EL solution setting up concentration from 20 mg/L to 60 mg/L was investigated. Each investigation was performed in triplicate for 24 h at room temperature. The residual supernatant EL concentrations were calculated by comparing the sample absorbance at 431 nm with the calibration curve.

Equations (1) and (2) (shown below) were used to calculate the removal efficiency (*R*,%) and biosorption capacity (*q_e_*, mg/g).

(1) $$R=\frac{C_{0}-C_{e}}{C_{0}}\cdot 100$$

(2) $$q_{e}=\frac{{(C}_{0}-C_{e})\cdot V}{m}$$

where *C_0_* and *C_e_* are EL initial and at-equilibrium concentrations (mg/L), *m* is the biosorbent dose (g/L), and *V* is the EL volume (L).

### 2.4. Modeling the Biosorption Experimental Data

#### 2.4.1. Kinetic Models

Adsorption kinetics determines the adsorption rate, which states the time necessary to achieve equilibrium in the adsorption process and is an important consideration in process development and adsorption system design. Kinetic models can help to understand the adsorption pathways and the likely mechanism that occurs [53]. 

Adsorption kinetic models were divided into two groups: adsorption reaction models and adsorption diffusion models. Adsorption reaction models reveal the rate of adsorbate adsorption by adsorbents but do not reveal the underlying reason for adsorption. On the other hand, mass action effect (adsorption/desorption between adsorbates and active sites of adsorbents) and external and internal (pore) diffusion are taken into account in adsorption diffusion models [54]. 

Adsorption employs either linear or nonlinear kinetic analysis. Kinetic models are typically subjected to accuracy testing utilizing model performance metrics (error functions) such as the coefficient of determination (R^2^) in order to find the model that best fits the experiment. The coefficient of determination, which indicates the variation from the mean, is used to assess the degrees of fit of kinetic models to experimental data [54]. 

Among the several current kinetic models, nonlinear forms of pseudo-first-order, pseudo-second-order, Elovich, Avrami and Weber–Morris were investigated to determine which ones were most suited to describe EL biosorption on LLA 5% biosorbent. Table 1 shows their individual equations, as well as the relevance of the parameters and measurement units.

The pseudo-first-order model is mostly used to examine adsorption data derived from aqueous solutions. It describes the adsorption rate, which is proportional to the number of available binding sites on the adsorbents [54]. The Lagergren pseudo-first-order model assumes that the rate of change in solute uptake over time is proportional to the difference in saturation concentration and solid uptake. This model is applicable to the initial stages of adsorption processes. When adsorption occurs by diffusion through the interface, the kinetics generally follow the Lagergren pseudo-first-order rate equation [53]. 

The pseudo-second-order kinetic model describes the adsorption of adsorbates onto adsorbents, in which the adsorbent’s adsorption capacity is determined by the chemical bonding (interaction) between adsorbates and functional groups on the adsorbent surface. It is based on equilibrium adsorption, which is determined by the amount of adsorbate adsorbed onto an adsorbent’s surface and the amount adsorbed at equilibrium. This model is used to forecast the order of the sorption process, as well as to evaluate the sorption capacity [54]. 

The Elovich model is used to describe adsorption processes that follow second-order kinetics under the premise that the adsorbent’s surface is energetically heterogeneous, resulting in various activation energies. It has been widely used in the study of chemisorption processes [54]. 

Weber and Morris proposed the intraparticle mass transfer diffusion model to identify the diffusion mechanism in the adsorption. If intraparticle diffusion plays a role in the adsorption process, *q_t_* vs. *t*^0.5^ plots should show straight lines. However, nonlinearity is occasionally found, indicating that various processes restrict the overall adsorption rate. If the results show multiline plots, it means that more than one process was involved in the adsorption [53].

#### 2.4.2. Equilibrium Isotherms

Adsorption isotherms describe the interaction of adsorbate and adsorbent and are critical for maximizing the utilization of any adsorbent [60]. The adsorption from the solution is assessed by measuring the decrease in the concentration of adsorbed species following absorption by solid. The adsorption isotherm is plotted as the amount adsorbed at equilibrium *q_e_* vs. the equilibrium concentration *C_e_* at constant temperature. The shape of an isotherm reveals the stability of adsorbent–adsorbate interactions as well as the molecules’ adsorption affinity. On the other hand, the adsorption isotherm represents the adsorbent’s main property, which is its ability to remove a specific species from the solution. Furthermore, each part of the adsorption curve can provide information regarding potential sorption mechanisms [53,60].

Many types of isotherm models have been used over the years, such as Langmuir, Freundlich, Redlich–Peterson, Dubinin–Radushkevich, Temkin, Hill, Toth and so on. Some rely on a simplified physical description of adsorption, whilst others are empirical and require experimental data correlation [53,60].

Among the isoterm models presented above, nonlinear forms of Freundlich, Redlich–Peterson, Temkin, Hill and Toth, were investigated to describe EL biosorption on LLA 5% biosorbent. Table 2 shows their individual equations, as well as the relevance of the parameters and measurement units.

Freundlich isotherm describes nonideal, multilayer, reversible adsorption at a heterogeneous surface, with the assumption that all adsorption sites have different binding energies. The energy distribution for adsorptive sites shows a spectrum of diverse binding energies rather than a single uniform energy and follows an exponential-type curve, which is similar to the real situation [53].

The Redlich–Peterson model developed an empirical model with three parameters to integrate the Langmuir and Freundlich approaches. This can depict adsorption equilibrium across a large concentration range, and because of its versatility, it may be used in both homogeneous and heterogeneous systems [53].

The Temkin model states that adsorption occurs in multiple layers and considers the effects of indirect adsorbate/adsorbate interactions on the adsorption process [66,67]. It is assumed that the heat of adsorption of all molecules in the layer falls linearly as surface coverage increases. The Temkin isotherm is only applicable to an intermediate range of ion concentrations, and extremely high and low concentration values of the adsorbate in the liquid phase are neglected [66,67].

The Toth isotherm is an empirical modification of the Langmuir equation designed to narrow the gap between experimental and anticipated equilibrium data. It assumes that the adsorption energies of most adsorption sites are less than the mean energy [66,67].

This model is best suited for describing heterogeneous adsorption systems that meet both the low- and high-end limits of adsorbate concentration [66].

The Hill isotherm equation describes the binding of various species onto homogenous substrates, assuming that adsorption is a cooperative phenomenon in which adsorbates at one site of the adsorbent influence different binding sites on the same adsorbent [66].

Several widely used kinetic models (pseudo-first-order, pseudo-second-order, Elovich, Avrami, Weber–Morris) and equilibrium isotherms (Freundlich, Redlich–Peterson, Temkin, Toth, Hill), from CAVS Adsorption Evaluation software version 2.0 were used to validate the biosorption of target pharmaceutical compound (Ethacridine lactate) by the biosorbent obtained by immobilization of *Lactococcus lactis* biomass in calcium alginate matrix.

### 2.5. Regeneration Studies

A number of desorption tests were conducted to evaluate the possibility of biosorbent regeneration. Five desorption eluents, including pure water, HCl (0.1 M and 0.05 M), HNO_3_ (0.1 M), NaOH (0.05 M and 0.1 M) and CH_3_COOH (0.1 M), were investigated. EL desorption from spent 5% LLA biosorbent was carried out in a batch system, using 1 g of biosorbent in contact with 25 mL of elution reagent for 24 h at room temperature. The outcomes that were determined were the desorption capacity of 5% LLA, which indicates the amount of EL desorbed (*q_des_*, mg of EL/g of 5% LLA) (Equation (3)) and the efficiency of the desorption process (*R_des_*%) (Equation (4)).

(3) $$q_{des}=\frac{C_{des}\cdot V}{m}$$

(4) $$R_{des}=\frac{C_{des}}{C_{abs}}\cdot 100$$

where *C_ads_* and *C_des_* (mg/L) are the concentrations of EL (adsorbed on 5% LLA and in the resulting solution after desorption), *m* is the amount of biosorbent loaded with EL (g), and *V* is the volume of the solution (L).

The supernatant EL concentrations were measured spectrophotometrically in the same manner as in the biosorption experiments.

## 3. Results and Discussion

### 3.1. Biosorbent Synthesis and Characterization

Natural polymers are nontoxic, biodegradable and readily available materials derived from renewable resources. Sodium alginate is a polymer formed from *β*-D-mannuronic acid (1–4) and *α*-L-guluronic acid. It is capable of building a network structure with divalent cations, such as calcium [68].

Taking into account the aforementioned abilities, its use resulted in the effective immobilization of *Lactococcus lactis* biomass, allowing us to create a new type of biosorbent.

**Figure 2:**
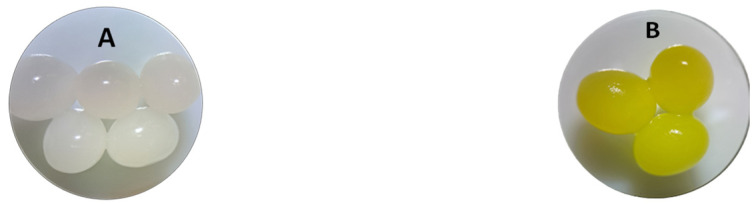
Photographs of synthesized biosorbent (LLA 5%) before biosorption (**A**) and after biosorption (**B**) of ethacridine lactate.

Figure 2 illustrates the aspect of the obtained beads (LLA 5%). As can be observed, the biosorbent beads exhibit a lighter shade of white and a regular, spherical shape, with a mean diameter of 3.073 ± 0.011 mm. 

**Figure 3:**
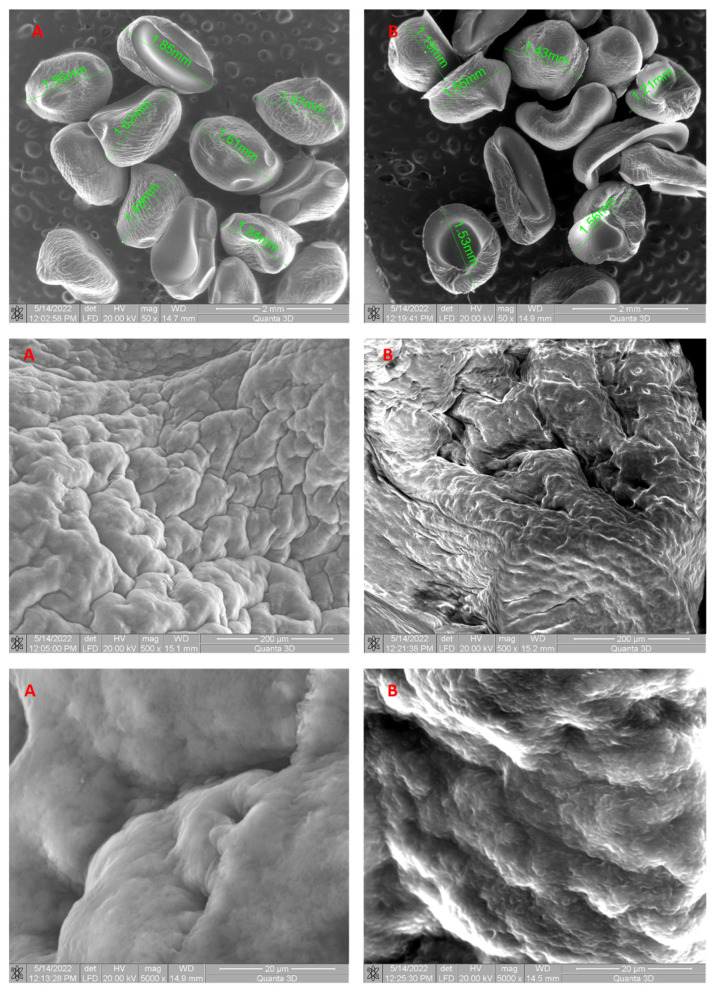
SEM images of 5% LLA biosorbent prepared before biosorption (**A**) and after biosorption (**B**) of ethacridine lactate from aqueous solution.

Scanning electron microscopy (SEM) was used to examine the morphological characteristics of the developed biosorbent. As several authors have stated, SEM is an effective and extensively used tool for investigating the morphological characteristics of adsorbent materials [29,69,70]. SEM images of the beads before and after ethacridine lactate biosorption from aqueous solutions employing LLA 5% as the biosorbent are shown in Figure 3. 

The irregular shapes of the grains observed in the SEM images are due to the fact that they were dried before the investigation was carried out. The synthesized LLA 5% beads before biosorption exhibit irregularly distributed pores of varying diameters, as seen by SEM images. The presence of pores favors the EL biosorption process both through a physical adsorption mechanism and by facilitating the contact between EL and the biosorbent. SEM images (Figure 3A,B) showed differences in the surface morphology of the beads. The surface texture of the 5% LLA granules changed after biosorption, indicating a higher propensity for agglomeration. Prior to biosorption, the 5% LLA granules exhibited a smoother surface with a certain porosity. The recorded modifications demonstrate that the tested contaminant was retained. The resultant granules are determined to be stable in terms of morphology following the biosorption process based on an analysis of the SEM pictures.

**Figure 4:**
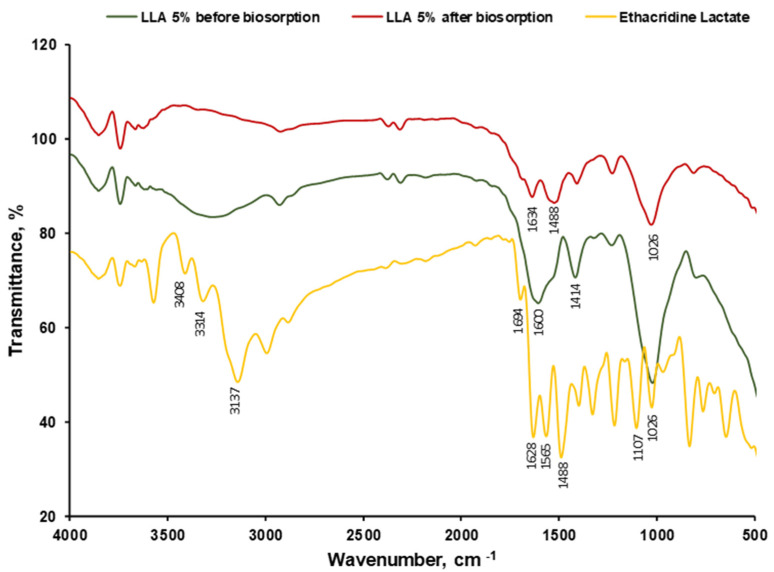
FTIR spectra of EL and 5% LLA biosorbent before and after ethacridine lactate biosorption.

FTIR spectra of 5% LLA before biosorption (Figure 4) showed the characteristic vibrational peaks of the alginate at 1012 cm^−1^ (C-O-C stretching), 1414 cm^−1^ (COO- symmetrical stretching), 1605 cm^−1^ (COO- asymmetrical stretching), 20863 cm^−1^ (C-H symmetrical stretching), 2926 cm^−1^ (aliphatic C-H asymmetrical stretching) and 3257 cm^−1^ (OH stretching). Similar assessments of alginate-based materials were presented by Pereira et al. [71]. The strong peak at 1565 cm^−1^ can be attributed to the N-H bending vibration coupled with the C-N stretching vibration of amide II. This confirms the presence of peptidoglycan from *L. lactis* bacteria (LAB) immobilized in the alginate matrix. It is known that the cell walls of LAB predominantly contain a peptidoglycan (also known as murein) that is formed by N-acetylglucosamine (NAG) and N-acetylmuramic acid (NAM) alternated in long chains and cross-linked by a tetrapeptide, composed of L-alanine, D-glutamine, L-lysine and D-alanine [72].

The vibrations with the maximum band width of about 1414 cm^−1^ are specific to C-OH bonds in the carbohydrate structure, which can be attributed to the presence of exopolysaccharides (EPS) produced by the *L. lactis* as mesophilic lactic acid bacteria [73,74,75].

When analyzing the two spectra, before and after biosorption, it is visible that some peaks of the ethacridine lactate and a few of the biosorbent’s functional groups are overlapping. Also, 5% LLA beads after biosorption present a higher transmittance value than before biosorption. This is encountered between 3100 cm^−1^ and 3500 cm^−1^ (frequency for N-H bond) where the signal for the OH group in the lactate (2500–3300 cm^−1^) overlaps the broad band at 3271 cm^−1^ (OH stretching). Besides this, a relatively strong peak recorded at 1628 cm^−1^ is assigned to the C=N stretching vibrations existing in the ethacridine lactate’s acridine ring. The peak at 1026 cm^−1^, which corresponds to the stretching vibration of the C-O bond in secondary alcohols, is evident in both the EL and LLA 5% spectra after biosorption, indicating that EL is retained on the biosorbent surface. The peak at 807 cm^−1^ indicates the presence of C_ar_-H out-of-plane deformation vibrations specific to ethacridine lactate [76].

The FTIR tests suggest that *L. lactis* bacteria immobilized in alginate establish possible interactions with ethacridine lactate, resulting in a potential for the biosorption of the pollutant by the 5% LLA beads.

**Figure 5:**
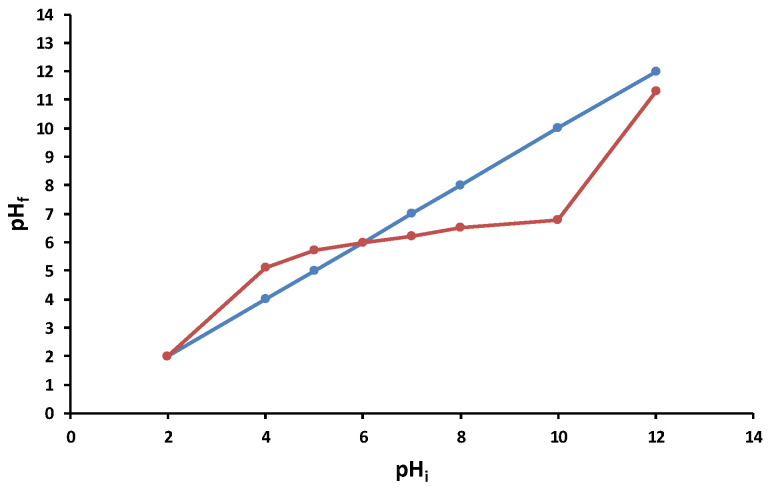
pH_PZC_ of 5% LLA biosorbent. (**—** tie line; **—** experimental data).

The point of zero charge for the 5% LLA biosorbent was determined to finish the characterization process (Figure 5). This point refers to the pH of a solution at which the charge of the positive and negative surface sites is equal, and hence the biosorbent surface charge is null. pH_PZC_ determines whether the surface charge is negative (pH > pH_PZC_) or positive (pH < pH_PZC_) [77]. 

As indicated in Figure 5, the pH_PZC_ of the 5% LLA biosorbent was determined at 6.00.

When pH_i_ is less than 5, the biosorbent surface is positively charged; when pH_i_ exceeds 10, it is negatively charged.

### 3.2. Influence of Main Parameters on the Biosorption Process

#### 3.2.1. Influence of pH on the Biosorption Process

The initial pH of the EL solution was chosen as the first parameter to explore how working conditions affect the biosorption process. In the current investigation, 10 mL of EL solutions with a concentration of 40 mg/L was mixed with biosorbents at a concentration of 2 g/L while the pH was changed from 2 to 12.

**Figure 6:**
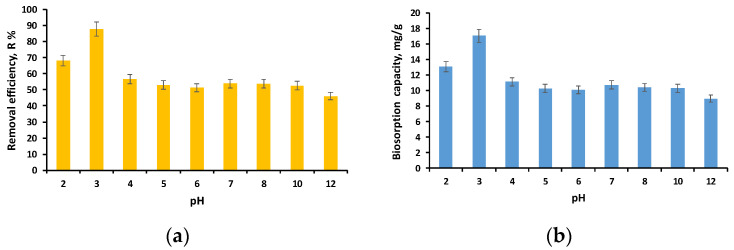
Influence of EL solution initial pH on removal efficiency (**a**) and biosorption capacity (**b**) (volume of EL solution: 10 mL; initial concentration of EL solution: 40 mg/L; biosorbent dose: 2 g/L; time: 24 h).

According to the data illustrated in Figure 6a,b, the same trend is observed for the removal efficiency as for the biosorption capacity. The lowest results are at pH 12, but similar for pH between 4 and 10. It should be mentioned that during the experiments, it was observed that at pH 2 and 12, deformations of the granules take place, and thus, at pH 2, the diameter decreases and at pH 12 increases as the swelling of the granules occurs.

These findings are congruent with the pH_PZC_ results, which showed that the EL solution had buffering properties between pH 4 and pH 10. The good retention of the ethacridine lactate at acidic pH can be explained by the fact that it is able to dissociate in aqueous solutions, and hence, it is adsorbed by the biosorbent’s positively charged surface. This aspect was confirmed both by this study and by a previous one [30]. 

Our premise is similar to that of Talman et al. [51], who determined that EL dissociates in aqueous solutions and that EL’s pH has little effect on the adsorption process. At pH 3, the highest reported removal efficiency and biosorption capacity were 87.84% and 17.06 mg/g, respectively. Thus, pH 3 was deemed appropriate for the future development of the biosorption process. Since the best results were obtained at pH 3, this may limit the process’s applicability. However, given that a concentration of 40 mg/L was tested and a removal efficiency of more than 50% was obtained for pH values ranging from 4 to 10 and that the concentrations of pharmaceutical compounds in aqueous matrices are micrograms or nanograms, we can consider the biosorbent effective in the pH range of 3 to 10.

#### 3.2.2. Influence of Biosorbent Dose on the Biosorption Process

**Figure 7:**
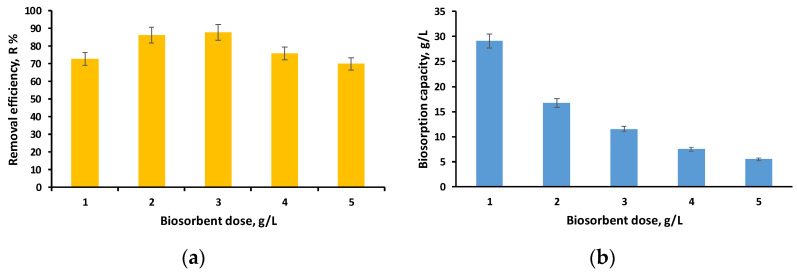
Influence of biosorbent dose on removal efficiency (**a**) and biosorption capacity (**b**) (volume of EL solution: 10 mL; initial concentration of EL solution: 40 mg/L; pH of initial EL solution: 3; time: 24 h).

In this step of our experimental study, we kept the pH and initial concentration of the EL solution constant while increasing the amount of biosorbent from 1 g/L to 5 g/L. Figure 7a,b illustrate the evolution of removal efficiency and biosorption capacity in the established settings.

A decrease in biosorption capacity is noted as the biosorbent dose increases, which is explained by the availability of more adsorption sites and increased removal efficiency (above 70%) for all doses investigated.

The best results related to the removal efficiency were obtained for the dose of 3 g/L (*R* = 87.67%, *q* = 11.53 mg/g), but considering the fact that for the dose of 2 g/L the results were similar (*R* = 86.16%, *q* = 16.73 mg/g), we considered that the difference of 1.51% cannot be considered significant enough to justify the use of a 50% higher biosorbent dose. As a consequence, a dose of 2 g/L was chosen for the subsequent tests. Our earlier investigation [52] yielded comparable results for two types of biosorbents made by immobilizing *Saccharomyces Pastorianus* biomass and residual *Saccharomyces Pastorianus* biomass.

#### 3.2.3. Influence of EL Initial Concentration on the Biosorption Process

The last variable that was examined to determine the impact of working parameters on the biosorption process was the initial concentration of the EL solution.

**Figure 8:**
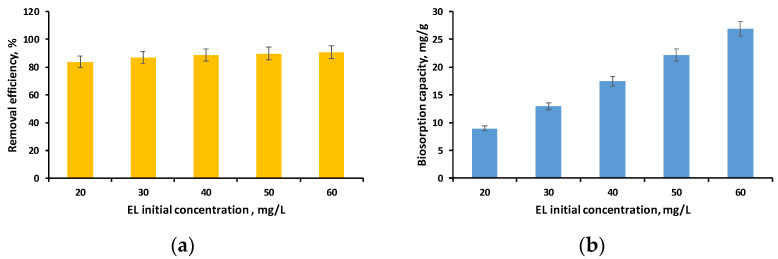
Influence of EL solution initial concentration on removal efficiency (**a**) and biosorption capacity (**b**) (volume of EL solution: 30 mL; pH of EL solution: 3; biosorbent dose: 2 g/L; time: 24 h).

Figure 8a,b show the evolution of removal efficiency and biosorption capacity when ethacridine lactate concentrations varied from 20 mg/L to 60 mg/L in the specifically defined conditions.

As can be seen in Figure 8a, the removal efficiency was higher than 80% for all the initial concentrations of EL tested, with the highest value of 90.72% being obtained in the case of the concentration of 60 mg/L. Similar findings were observed in our earlier investigation where we achieved a removal efficiency of more than 90% for EL concentrations ranging from 20 to 60 mg/L using biosorbents obtained by immobilizing *Saccharomices Pastorianus* biomass (inactivated or residual) in calcium alginate [52].

This leads to the conclusion that the synthesized biosorbent can be used to retain the tested pharmaceutical compound from aqueous solutions with good results over a wide range of concentrations, with relatively high values when considering the fact that pollutants of this type are found in wastewater at microgram levels.

### 3.3. Kinetic Evaluation of the Biosorption Process

Kinetics experiments were performed in batch mode at pH 3, with EL volumes of 30 mL and biosorbent doses of 2 g/L, with initial concentrations of EL ranging from 20 mg/L to 60 mg/L. Samples were taken at different time intervals and analyzed.

Among the various kinetic models available in the literature [53,54,60,78], the nonlinear forms of pseudo-first-order, pseudo-second-order, Elovich, Avrami and Weber–Moris models were investigated to identify which are most appropriate for describing EL biosorption on the 5% LLA biosorbent at previously established working parameters.

**Figure 9:**
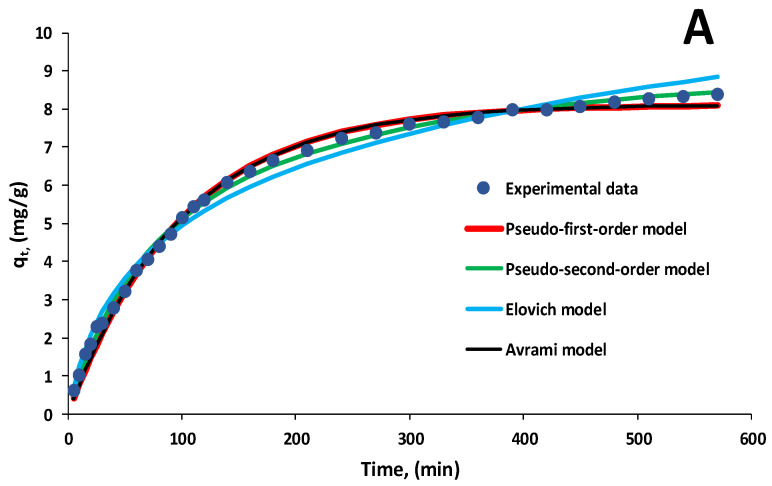

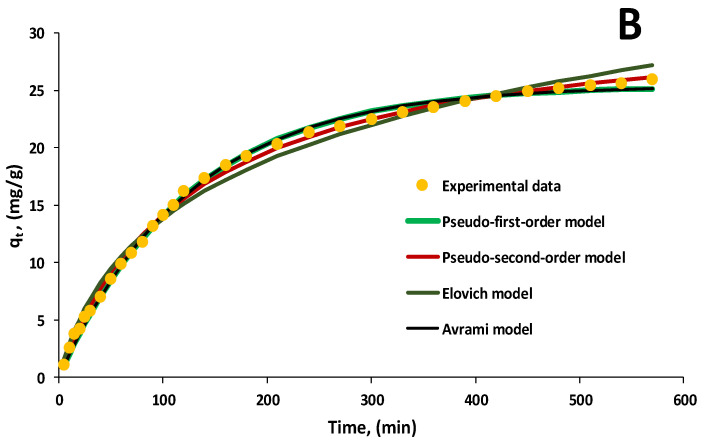
Kinetic models for biosorption of EL on synthesized biosorbent 5% LLA ((**A**)—initial concentration of EL solution 20 mg/L; (**B**)—initial concentration of EL solution 60 mg/L).

Figure 9A,B exemplify the aforementioned kinetic models adapted to the experimental data recorded in the case of biosorption performed with 30 mL of EL solution having pH 3 and a biosorbent dose of 2 g/L for two initial pollutant concentrations of 20 mg/L and 60 mg/L. The contact time was set to 570 min.

Table 3 shows the kinetic characteristics of the biosorption process as determined by model plots for all concentrations examined.

The pseudo-first-order, pseudo-second-order and Avrami models are the most comparable to the experimental data. From these, the pseudo-second-order model provides the greatest fit together with correlation coefficients better than 0.991. The aforementioned results suggest that chemical biosorption occurred as a result of interactions between EL and functional groups on the biosorbent surface, which influenced biosorption rates.

Silva et al. [79] made a similar observation when studying the adsorption of ibuprofen and acetaminophen on magnetic beads of alginate/polypyrrole/ZnFe_2_O_4_ and concluded that the pseudo-second-order kinetic model is best suited for describing the interactions between the adsorbent and the tested drugs.

Amor et al. [80] show that the kinetic outcomes fit the pseudo-second-order model for the adsorption of the cytostatic drug 5-fluorouracil (5-FU) from water samples onto alginate/geopolymer hybrid beads (AGHB) in the static regime.

The authors explain this kinetic behavior by stating that, in addition to the pore-filling sorption process, the carboxyl and hydroxyl groups of alginate immobilized on the geopolymer matrix promoted the retention of 5-FU ions via hydrogen bonding [80].

Table 3 shows that the constant *k_2_* varies with experimental circumstances, particularly the starting concentration of the pollutant in the aqueous solution, and correlates with R^2^.

The lower value of *k_2_* for an initial EL concentration of 60 mg/L suggests that the time necessary to attain equilibrium is relatively long. The quantity of contaminant retained at equilibrium (*q_e_*) is the second most important parameter in the pseudo-second-order kinetic model. There were no significant differences between the experimental results and the mathematical model’s predictions for any of the concentrations that were tested.

The Elovich kinetic model is known to be quite close to that stated by the pseudo-second-order model [54]; however, in our instance, the correlation coefficients were lower for this model, despite the fact that values greater than 0.981 were achieved for all concentrations.

In the Avrami model, the constant *n_Av_* has values less than one, indicating that biosorption is homogenous and does not evolve at a constant rate. The authors obtained similar results in their previous study for the biosorption of pharmaceutical compounds on a biocomposite material obtained by immobilizing *Saccharomyces pastorianus* on calcium alginate [52].

### 3.4. Equilibrium Isotherms

To understand the biosorption mechanisms of EL on the synthesized biosorbent, the obtained experimental data were analyzed using the following equilibrium isotherms: Freundlich, Redlich–Peterson, Temkin, Hill and Toth.

**Figure 10:**
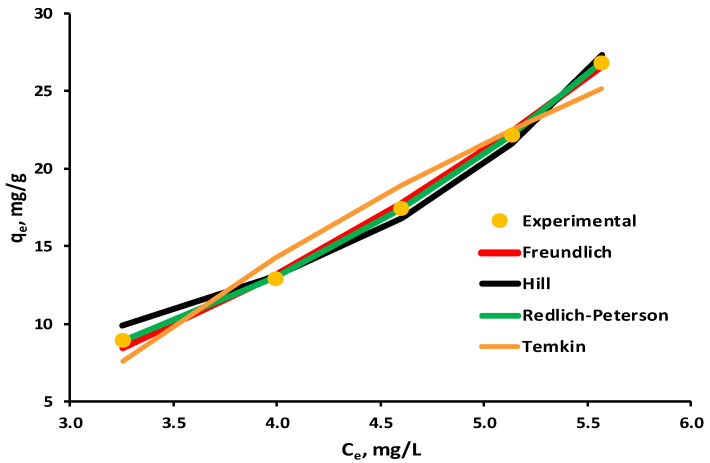
Equilibrium isotherms for biosorption of EL on synthesized biosorbent 5% LLA.

A graphical representation of the biosorption isotherms investigated in relation to the experimental data is presented in Figure 10, with the constraint that only models with a correlation coefficient R^2^ greater than 0.95 were included.

The parameters of tested equilibrium isotherms are given in Table 4. The correlation coefficient R^2^ for the Toth adsorption isotherm was 0.7526, indicating that the experimental data do not fit with this model, and hence its results are not included in the table.

According to correlation coefficient values, four of them, in the order of Redlich–Peterson > Freundlich > Hill > Temkin, are quite pertinent to describe the experimental data for EL biosorption on the 5% LLA biosorbent.

The result obtained for the Freundlich isotherm (R^2^ = 0.9978) leads to the hypothesis that the biosorption process occurs as multilayer adsorption on a heterogeneous surface.

This is supported by the validation of the Redlich–Peterson model with a correlation coefficient of 0.9999, given that it is employed in both homogeneous and heterogeneous adsorption.

The Temkin model ignores both high and low concentrations of the adsorbate in the liquid phase, and it also assumes that adsorption occurs in multilayers. In the current situation, smaller values of the *b* Temkin constant indicate that the interactions between the biosorbents and the target molecule are weak, which supports physisorption.

A negative result for *n_hi_* was obtained employing the Hill isotherm, which takes into account the adsorbate’s ability to bind to one site of the biosorbent while influencing the other sites, suggesting negative cooperativity.

The results differ from those obtained in our previous study for pharmaceutical biosorption using the *Sacharomyces pastorianus*/calcium alginate matrix as a biosorbent, where the models that were fairly pertinent to describe the experimental data followed the sequence Sips = Hill > Freundlich > Redlich–Peterson > Temkin > Toth [52].

Correa-Navarro et al. [81] used biochar from fique bagasse to remove caffeine and diclofenac from an aqueous solution and concluded that the Redlich–Peterson isotherm model fitted very well the experimental data for both pollutants.

On the contrary, in another study [82], the authors demonstrate that the Alizarin Red S dye adsorption on *Aspergillus terreus*/ sodium alginate composite beads was well explained by the Langmuir isotherm. Another study conducted for the removal of congo red and coralene dark red 2B dyes from aqueous solutions by adsorption on sodium alginate-based composite films established that the adsorption mechanism was confirmed through the Langmuir isotherm [83].

Silva et al. [79] state that in the case of ibuprofen and acetaminophen adsorption on alginate/polypyrrole/ZnFe_2_O_4_ magnetic beads, the adsorption mechanism was fitted by the Freundlich isotherm.

### 3.5. Regeneration and Reusability of Spent 5% LLA Biosorbent

Regeneration of the biosorbent is an essential phase in boosting the biosorption system’s efficiency and restoring the sorbent’s commercial viability. Desorption is a method for recycling and reusing biosorbents by retrieving the adsorbed pharmaceutical compound. In this context, a series of preliminary investigations were made, the results being presented in Table 5.

The results reported above indicate that 0.05 M HCl could be a better reagent for desorption as well as the possibility of efficient EL desorption.

Further research is needed to optimize the regeneration, understand the desorption mechanism, establish the number of regeneration cycles and identify the techno-economic circumstances under which they are effective. In the tests shown in Table 5, it should be noted that the solution-to-biosorbent mass ratio was kept constant, which affected the *R_des_*% but did not necessitate more solution than was treated in the biosorption experiments. The ability to reuse 5% LLA in a new biosorption process is dependent not only on *R_des_*% but also on the amount of eluent.

## 4. Conclusions

The current study presents the synthesis of a new biosorbent by immobilizing Lactococcus lactis (a lactic bacteria) in a calcium alginate matrix and its performance in biosorption processes to remove a selected target pharmaceutical compound (EL) from aqueous solutions.

The synthesized biosorbent was investigated for morphology (SEM), functional groups (FTIR), particle size and the point of zero charge. The obtained beads named 5% LLA exhibit a lighter shade of white and a regular, spherical shape, with a mean diameter of 3.073 ± 0.011 mm. The recorded SEM images before and after biosorption demonstrate that the tested contaminant was retained. The analysis of the FTIR spectra before and after biosorption suggests that *L. lactis* bacteria immobilized in alginate establish possible interactions with ethacridine lactate, thus resulting in a potential for pollutant biosorption by the 5% LLA granules. The value for pH_PZC_ was established at 6.00.

Batch biosorption studies were conducted to determine the effect of the main biosorption process parameters, including initial pH, biosorbent amount and EL initial concentration. For all EL initial concentrations tested, a removal efficiency of more than 80% was achieved with an initial pH of 3.0 and a biosorbent dose of 2 g/L, while the highest value of 90.72% was obtained in the case of the concentration of 60 mg/L. Given that the best results were obtained at pH 3 (*R* = 87.84%), the process’s applicability may be limited; however, given that pharmaceutical concentrations in water are in the order of g and ng/L, and a removal efficiency of more than 50% was obtained for a concentration of 40 mg/L for the pH range of 4 to 10, we can consider the biosorbent effective in the pH range of 3 to 10.

The kinetics approach involved assessing nonlinear versions of pseudo-first-order, pseudo-second-order, Elovich, Avrami and Weber–Moris models to determine which are most appropriate for characterizing EL biosorption on the 5% LLA biosorbent at defined operating parameters. Apart from the examined models, the pseudo-second-order model provides the most accurate fit together with correlation coefficients better than 0.991.

In this study, several models were applied to assess and fit the experimental data, taking into account the importance of equilibrium isotherms in the practical design of biosorption processes. According to correlation coefficient values, four of them, in the order of Redlich–Peterson > Freundlich > Hill > Temkin, are quite pertinent to describe the experimental data for EL biosorption on the 5% LLA biosorbent.

Finally, compared to other conventional adsorbents, the 5% LLA biosorbent is an innovative composite that can be employed as an inexpensive, sustainable, easily manufactured and eco-friendly adsorbent. The main advantage of this material is that it can be synthesized from available and renewable resources. It should be highlighted that the 5% LLA biosorbent could be regenerated with a 0.05 M hydrochloric acid solution, which provides a significant benefit in biosorption processes. To apply this type of biocomposite material at an advanced technological level, investigations of the biosorption process in a dynamic system and testing on different types of aqueous matrices, including wastewater treatment plant effluents, are required.

## Figures and Tables

**Table 1:** Nonlinear equations of tested kinetic models.

Kinetic Model	Equation	Parameter Significance	Ref. *
Pseudo-first-order	$q_{t}=q_{e}\cdot (1-e^{-k_{1}\cdot t})$	*t*—time (min), *q_t_*—concentration on the solid phase at time *t* (mg/g), *q_e_*—adsorbent capacity at equilibrium (mg/g), *k*_1_—constant rate (L/min)	[55]
Pseudo-second-order	$q_{t}=\frac{k_{2}\cdot q_{e}^{2}\cdot t}{\left(1+k_{2}\cdot q_{e}\cdot t\right)}$	*t*—time (min), *q_t_*—concentration on the solid phase at time *t* (mg/g), *q_e_*—adsorbent capacity at equilibrium (mg/g), *k*_2_—constant rate (L/min)	[56]
Elovich	$q_{t}=\frac{1}{\beta }\cdot ln(1+\alpha \cdot \beta \cdot t)$	*t*—time (min), *q_t_*—concentration on the solid phase at time *t* (mg/g), *α*—initial adsorption rate (mg/(g·min)), *β*—extent of surface coverage and activation energy for chemisorption (g/mg)	[57]
Avrami	$q_{t}=q_{e}\cdot (1-e^{\left(-k_{Av}\cdot t^{n_{Av}}\right)})$	*t*—time (min), *qt*—concentration on the solid phase at time *t* (mg/g), *q_e_*—adsorbent capacity at equilibrium (mg/g), *k_avr*—the overall rate constant (L/min), *n_avr*– (dimensionless) is parameter related to adsorption	[58]
Weber–Morris	$q_{t}=k_{wm}\cdot t^{0.5}+B$	*t*—time (min), *qt*—concentration on the solid phase at time *t* (mg/g), *K_wm_—*(mg/g·min^0.5^), *B* –provides information about the thickness of the boundary layer (mg/g)	[59]

Ref. *—first reference for each mentioned model.

**Table 2:** Nonlinear equations of investigated equilibrium isotherms.

Equilibrium Isotherm	Equation	Constant Significance	Ref. *
Freundlich	$q_{e}=K_{f}\cdot {C_{e}\;}^{\frac{1}{n}}$	*q_e_*—adsorbate concentration on the solid phase at equilibrium (mg/g), *C_e_*—adsorbate concentration on the fluid phase at equilibrium (mg/L), *K_f_*—Freundlich constant ((mg/g)$({L/mg)}^{\frac{1}{n}}\;),$ *n*—Freundlich constant (dimensionless)	[61]
Redlich–Peterson	$q_{e}=\frac{K_{r1}\cdot C_{e}}{1+K_{r2}\cdot C_{e}^{n}}$	*q_e_*—adsorbate concentration on the solid phase at equilibrium (mg/g), *C_e_*—adsorbate concentration on the fluid phase at equilibrium (mg/L), *K_r1,,_ K_r2_*—Redlich–Peterson constants, *C_e_^n^*—Redlich–Peterson exponent (dimensionless)	[62]
Temkin	$q_{e}=\frac{R\cdot T}{b}\cdot ln(K_{t}\cdot C_{e})$	*q_e_*—adsorbate concentration on the solid phase at equilibrium (mg/g), *C_e_*—adsorbate concentration on the fluid phase at equilibrium (mg/L), *R*—gas constant (*R* = 8.314 J/(mol K)), *T*—temperature (K), *K_t_*—Temkin constant (L/mg), *b*—Temkin constant (J/mg)	[63]
Toth	$q_{e}=\frac{q_{t}\cdot C_{e}}{{\left(b_{t}+C_{e}^{n_{t}}\right)}^{n_{t}}}$	*q_e_*—adsorbate concentration on the solid phase at equilibrium (mg/g), *C_e_*—adsorbate concentration on the fluid phase at equilibrium (mg/L), *q_t_*—Toth maximum uptake (mg/g), *b_t_*—Toth constant (L/mg), *n_t_*—Toth constant (dimensionless)	[64]
Hill	$q_{e}=\frac{q_{h}\cdot C_{e}^{n_{h}}}{K_{h}\cdot C_{e}^{n_{h}}}$	*q_e_*—adsorbate concentration on the solid phase at equilibrium (mg/g), *C_e_*—adsorbate concentration on the fluid phase at equilibrium (mg/L), *q_h_*—Hill maximum uptake (mg/g), *K_h_*—Hill constant (L/mg), *n_h_*—cooperativity coefficient of the binding interaction (dimensionless)	[65]

Ref. *—first reference for each mentioned model.

**Table 3:** Kinetic parameters of EL biosorption process on 5% LLA biosorbent.

Kinetic Model	EL Initial Concentration, mg/L	*q_e_*	*k_1_*	*k_2_*	*α*	*β*	*k* *_Av_*	*n* *_Av_*	*k*	*B*	R^2^
Pseudo-first- order	20	8.1175	0.0101	-	-	-	-	-	-	-	0.9939
	30	12.2177	0.0082	-	-	-	-	-	-	-	0.9980
	40	16.3806	0.0109	-	-	-	-	-	-	-	0.9908
	50	20.8372	0.0090	-	-	-	-	-	-	-	0.9949
	60	25.3984	0.0081	-	-	-	-	-	-	-	0.9972
Pseudo-second-order	20	9.8225	-	0.0011	-	-	-	-	-	-	0.9976
	30	15.4417	-	0.0005	-	-	-	-	-	-	0.9942
	40	19.4463	-	0.0006	-	-	-	-	-	-	0.9911
	50	25.7185	-	0.0004	-	-	-	-	-	-	0.9977
	60	31.9761	-	0.0002	-	-	-	-	-	-	0.9981
Elovich	20	-	-	-	0.1648	0.4180	-	-	-	-	0.9873
	30	-	-	-	0.1618	0.2386	-	-	-	-	0.9818
	40	-	-	-	0.4205	0.2246	-	-	-	-	0.9897
	50	-	-	-	0.3430	0.1524	-	-	-	-	0.9896
	60	-	-	-	0.3458	0.1170	-	-	-	-	0.9904
Avrami	20	8.1175	-	-	-	-	0.1481	0.6793	-	-	0.9939
	30	12.2177	-	-	-	-	0.0093	0.8753	-	-	0.9979
	40	16.3629	-	-	-	-	0.0117	0.9365	-	-	0.9875
	50	20.8372	-	-	-	-	0.0115	0.7751	-	-	0.9949
	60	25.3984	-	-	-	-	0.0088	0.9217	-	-	0.9972
Weber–Morris	20	-	-	-	-	-	-	-	0.3622	0.8553	0.9284
	30	-	-	-	-	-	-	-	0.5835	0.1741	0.9351
	40	-	-	-	-	-	-	-	0.6954	2.5525	0.9392
	50	-	-	-	-	-	-	-	0.9507	1.4244	0.9438
	60	-	-	-	-	-	-	-	1.1861	0.7804	0.9517

**Table 4:** Equilibrium isotherm parameters of biosorption process conducted on 5% LLA biosorbent.

Parameter	Freundlich	Temkin	Hill	Redlich–Peterson
*K_fr_*	0.6929	-	-	-
*n_fr_*	0.4709	-	-	-
*K_t_*	-	0.3871	-	-
*b_t_*	-	75.0691	-	-
*q_hi_*	-	-	0.0020	-
*K_hi_*	-	-	−0.9995	-
*n_hi_*	-	-	−0.0002	-
*K_rd_*_1_	-	-	-	0.3349
*K_rd_*_2_	-	-	-	−0.7713
*n_rd_*	-	-	-	0.1094
R^2^	0.9978	0.9563	0.9907	0.9999

**Table 5:** Results of preliminary investigations for regeneration of spent 5% LLA biosorbent.

Desorbtion Reagent, Concentration	*q_ads_* * (mg/g)	*q_des_* (mg/g)	*R_des_* (%)
Distilled H_2_O	17.3235	-	-
0.1 M HCl	17.3235	7.9913	46.9624
0.05 M HCl	17.3235	9.7217	56.9913
0.1 M CH_3_COOH	17.3235	1.5956	9.3539
0.1 M HNO_3_	17.3235	3.0103	17.6471
0.1 M NaOH	17.3235	Desorption in strongly basic environments causes irreversible damage to the polymer matrix and distortion of the granule structure due to swelling or rupture.	
0.05 M NaOH	17.3235		

* represents biosorption capacity of 5% LLA achieved under batch conditions by contacting 10 g of 5% LLA with 250 mL of EL solution with initial concentration of 40 mg/L at pH 3 for 24 h at room temperature.

## Data Availability

The original contributions presented in the study are included in the article, further inquiries can be directed to the corresponding author.
